# Technical procedures for template-guided surgery for mandibular reconstruction based on digital design and manufacturing

**DOI:** 10.1186/1475-925X-13-63

**Published:** 2014-05-23

**Authors:** Yun-feng Liu, Liang-wei Xu, Hui-yong Zhu, Sean Shih-Yao Liu

**Affiliations:** 1 Key Laboratory of E&M (Zhejiang University of Technology), Ministry of Education & Zhejiang Province, Hangzhou 310014, China; 2 Department of Stomatology, The First Affiliated Hospital, School of Medicine, Zhejiang University, Hangzhou 310006, China; 3 Department of Orthodontics and Oral Facial Genetics, School of Dentistry, Indiana University, Indianapolis 46202, IN, USA

**Keywords:** Template-guided sugery, Mandibular reconstruction, Virtual planning, 3D printing

## Abstract

**Background:**

The occurrence of mandibular defects caused by tumors has been continuously increasing in China in recent years. Conversely, results of the repair of mandibular defects affect the recovery of oral function and patient appearance, and the requirements for accuracy and high surgical quality must be more stringent. Digital techniques — including model reconstruction based on medical images, computer-aided design, and additive manufacturing — have been widely used in modern medicine to improve the accuracy and quality of diagnosis and surgery. However, some special software platforms and services from international companies are not always available for most of researchers and surgeons because they are expensive and time-consuming.

**Methods:**

Here, a new technical solution for guided surgery for the repair of mandibular defects is proposed, based on general popular tools in medical image processing, 3D (3 dimension) model reconstruction, digital design, and fabrication via 3D printing. First, CT (computerized tomography) images are processed to reconstruct the 3D model of the mandible and fibular bone. The defect area is then replaced by healthy contralateral bone to create the repair model. With the repair model as reference, the graft shape and cutline are designed on fibular bone, as is the guide for cutting and shaping. The physical model, fabricated via 3D printing, including surgical guide, the original model, and the repair model, can be used to preform a titanium locking plate, as well as to design and verify the surgical plan and guide. In clinics, surgeons can operate with the help of the surgical guide and preformed plate to realize the predesigned surgical plan.

**Results:**

With sufficient communication between engineers and surgeons, an optimal surgical plan can be designed via some common software platforms but needs to be translated to the clinic. Based on customized models and tools, including three surgical guides, preformed titanium plate for fixation, and physical models of the mandible, grafts for defect repair can be cut from fibular bone, shaped with high accuracy during surgery, and fixed with a well-fitting preformed locking plate, so that the predesigned plan can be performed in the clinic and the oral function and appearance of the patient are recovered. This method requires 20% less operating time compared with conventional surgery, and the advantages in cost and convenience are significant compared with those of existing commercial services in China.

**Conclusions:**

This comparison between two groups of cases illustrates that, with the proposed method, the accuracy of mandibular defect repair surgery is increased significantly and is less time-consuming, and patients are satisfied with both the recovery of oral function and their appearance. Until now, more than 15 cases have been treated with the proposed methods, so their feasibility and validity have been verified.

## Background

The mandibular bone is one of the major components of the temporomandibular joint (TMJ). Defects of the mandible, typically caused by tumors such as ameloblastoma, often lead to severe facial deformity and difficulty with chewing which, in turn, significantly affect a patient’s quality of life. Reconstruction of the mandibular defect has been performed by various techniques including iliac bone grafts, costochondral grafts, a sliding vertical osteotomy on the posterior border of the mandibular ramus, sternoclavicular grafts, scapular flaps [1], and vascularized second metatarsal joint grafts [2]. Hidalgo introduced the vascularized fibular graft, which became the gold standard for the reconstruction of the mandibular condyle [3], because it offers several advantages: it can supply a large amount of bone and soft tissue for harvest [4]; the vessel pedicle is generally long and anatomically reliable; furthermore, this procedure involves only minor donor site morbidity, with no requirement for patient repositioning during surgery [5].

For restoration of the patient’s appearance and oral function, the fibula graft should be redesigned and shaped. For ideal preparation of the graft replacing the defect, to merge well with remaining bone, the resecting curves of the defect and the border of the graft for repair should also be designed [6]. In China, however, most mandibular reconstruction surgeries are performed by conventional methods, “free hand,” relying mainly on surgeons’ experience, 2D x-ray imaging, and some simple measuring tools [7]. Decades ago, advanced engineering technology — including medical image processing, computer-aided design (CAD), computer-aided manufacturing (CAM), additive manufacturing (AM, formerly known as rapid prototyping or RP, now widely known as 3D printing), and digital design based directly on a triangular mesh model — has been widely used in medical research [8], especially in maxillofacial surgery, including orthognathic surgery [9], dental implants [10,11], apicoectomy [12], and mandibular reconstruction [13-15]. With these advanced techniques, surgeons can precisely diagnosis the defect in a 3D environment, predesign a surgical plan, and even perform the plan in the clinic with the help of customized tools such as a surgical guide, thereby significantly improving the quality and efficiency of the surgery.

These advanced engineering techniques have been used in some cases for mandibular reconstruction [16]. Guided dental implantation in mandibles reconstructed with a free fibular flap has been studied and performed clinically, with the help of CT, a predesigned plan, and a surgical template, whereby the surgeon can overcome the difficulties encountered in dental implanting after fibular graft surgery [17,18]. Even with the help of navigation systems both for surgeries of dental implanting and mandibular reconstruction, the mandible repair and dental prosthesis with immediate loading can be realized in one operational process, which can improve the patient’s postoperative feel on aesthetic appearance and oral function significantly [19]. Among these reported applications, most of them aimed to design a surgical plan and fabricate the physical model of bone, but in the clinic, these models were used only as reference rather than as a surgical guide, because no templates for tumor resection, graft harvesting, and shaping were designed and fabricated, so the advanced engineering technology has not yet taken full effect in the clinic [20]; and also, some of these existing application research presented full accurate solution for mandible reconstruction involving tumor resection, fibula graft osteotomy, shaping and implanting, based on some commercial services, which can provide surgical templates for the four operations, such as ProPlan CMF ^TM^ and SurgiCase^TM^ (Materialse NV, Leuven, Belgium) [21], and these solutions have been proved with high accuracy and less postoperative complications [22]. But the survey data from clinics in China also proves that surgical templates are rarely used during operation for mandibular reconstruction, and the virtual plan is only used at preoperative phase and kept in the computer [23]. The main reasons include the facts that the price of these services is too high, and the template is time-consuming to acquire, because it is fabricated overseas. Furthermore, this service has not been certified in China, so it is only used for some research requirements.

To transfer the virtual plan to clinical operation, the resection of tumor, the osteotomy of fibular bone, the shape of graft, and the shape of fixation plate should be all controlled accurately by four templates, but rarely realized in presented research, among which, only one or two templates have been involved [24], because the commercial systems are not always available for most researchers and dentists. Aiming at this problem, based on general platforms which can be acquired conveniently, this study presents a new technical solution for guided surgery of mandibular reconstruction with a fibular flap. From 15 cases, taking a typical case as an example, the key techniques — including medical image processing, plan of tumor resection, fibular resection for graft harvesting, graft shaping, and fixation, as well as design and fabrication of the templates for clinical realization of the plan — are discussed in detail.

## Patients and methods

### Patients

During the past two years, we have successfully used templates guided surgery of fibular free flaps for mandibular reconstruction with a solution based on general platforms on 15 cases (Table 1). These cases consisted of 8 males and 7 females with an average age of 39.8 years (rage 15–63 years). These patients were treated by one surgical team between December 2011 and December 2013. The defects of tumor include ameloblastoma, fibroma and gingival carcinoma with size between 3 cm × 3 cm-10 cm × 5 cm, and the size of the flaps which were used for reconstruct defects ranged between 9.5 cm and 17 cm.

**Table 1:** Details of patients

**Variables**	**Numbers**	**Percentage**
Ages		
<30	4	27%
30-50	7	46%
>50	4	27%
Sex		
Male	8	54%
Female	7	46%
Diagnosis (Defect lesion type)		
Ameloblastoma	7	46%
Fibroma	4	27%
Gingival carcinoma	4	27%
Mandibular defect type		
Lateral	7	46%
Lateral-central	3	20%
Lateral-central-lateral	5	34%

### Technical procedure for surgical plan design and realization

Free vascularized bone transfer has become a preferred method for the reconstruction of mandibular defects, following resection of advanced cancers in the oral cavity. For successful reconstruction, careful preoperative evaluation and planning, proper flap-harvesting techniques, and appropriate manipulation and flap shaping with minimal vascular damage are essential steps for the recovery of mandibular function, mastication, and aesthetic appearance. Currently, the standard method of vascularized bone transfer involves fixation with titanium reconstruction plates or miniplates. When the reconstruction plate is bent to fit the native mandible, it acts as a 3D template for reconstruction [25].

**Figure 1:**
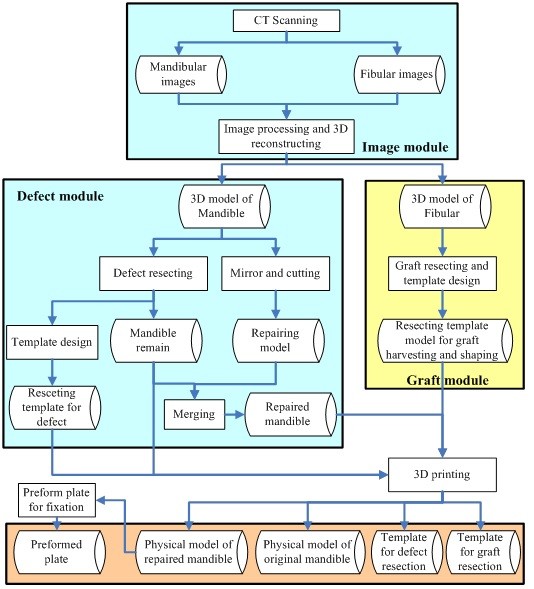
**Outline of technical procedure.** Three modules: image processing, defect resection, and graft osteotomy, are included.

A conventional surgical plan for mandibular defect and fibular graft resection is based on diagnosis via 2D images and measurement in the clinic, and can be performed by a dentist with general tools and experience. Obviously, the area for resection cannot be positioned precisely, nor can the graft be shaped ideally, so surgical time and risk are increased.The technical procedure is outlined in Figure 1. According to the surgical plan and template, the procedure is divided into three main modules: image, defect, and graft. Two fabrication steps, 3D printing and fixation plate preforming, are not commonly included in the three moduli. First, CT images, including mandibular and fibular sites, are acquired and processed in an image module, and 3D mandibular and fibular models are reconstructed and exported as stereolithography (STL) files. The mandibular model is then processed in the defect module, which includes two main functions, one to create a resection template for the defect, and the other to generate a repaired model for the preforming reconstruction plate. For design of a defect resection template, the tumor defect area is outlined under the direction and supervision of the surgeon, and the original model is then separated into the defective and healthy parts. Based on resecting curves and the original model, the resection template is designed via mesh offset. The healthy area is prepared to merge with the area to be repaired, which is separated from the contralateral model, creating the repaired model.

The templates for fibular resection, graft harvesting, and graft shaping are designed with several steps in the graft module. The length and site of the fibular graft are determined by defect size, so the fibula is surgically simulated for resection and shaping on the mandibular model. Based on the surgical plan design and simulation, the final model of the vascularized fibular graft is acquired, then used to design a resection and shaping template to transfer the virtual plan from computer to clinic.As described in Figure 1, the data for the 3D model of bone and templates are imported into a 3D printing machine, and 4 physical sections are fabricated: the original and repaired mandibular models, the template for defect resection, and the templates for fibular graft resection and shaping. The titanium reconstruction plate for graft fixation is blended and performed on the repaired model and can be used as a graft-positioning template in the defect removal area.

To present this technical procedure conveniently in detail, we selected a typical case of a female patient (27 years of age) requesting diagnosis of a continuously increasing lump on the left mandible, first identified 6 months previously. Checking by hand confirmed the presence of a golf-ball-sized lump in the left mandible, measuring 3 cm × 3 cm, tough, stable, and with clear borders. The lump was diagnosed further, with panoramic x-ray and tissue slice, as an ameloblastoma, and surgical treatment with vascularized fibular flap transfer was proposed. In this case, with the fabricated models, the virtual plan was realized competently and efficiently in the clinic. During the plan design, some parameters should be cared for and evaluated carefully, including the size of tumor, the length of fibular graft, and the shape of graft. These parameters will determine the restoration on oral function and appearance, including speech, mastication, mouth open, as well as appearance symmetry.

### Medical image acquisition

To reconstruct the mandible with an autograft, two surgeries are needed, one at the defect site, the other at the donor site. Hence, accurate mandibular reconstruction with a fibular flap requires 2 CT scans, one for the mandible, the other for the fibular flap. Both CBCT (cone-beam CT) for dentistry and general spiral CT are suitable, but for spiral CT, the interval between two slices should be smaller than 1 mm to guarantee a precisely reconstructed model.

In this case, the scan was carried out on a helical CT scanner (Brilliance CT 64-channel, Philips Healthcare, Best, Netherlands) under the following conditions: 120 kV, 250 mA, 1-mm slice thickness, 0.5-mm slice interval, 0.75-s rotation time, and 512 × 512 image resolution. The images of the maxillofacial region (312 images) and the fibular region (585 images) were separately recorded on a disc in a DICOM format (Digital Imaging and Communications in Medicine) file.

### Image processing and 3D medical model reconstruction

**Figure 2:**
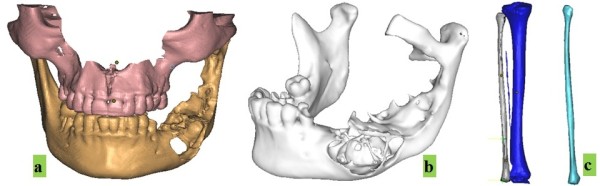
**Reconstructed mandibular and fibular models. (a)** Reconstructed maxilla and mandible. **(b)** Separated mandible shows the defect. **(c)** Reconstructed shank bones and separated fibular bone.

The images are imported and processed with medical software of a popular commercial platform, Mimics (version 11.04, Materialise NV). With this tool, the images are processed for reconstruction of a 3D model of bone in three steps: threshold of the Hounsfield value setting, growing region, and 3D model calculation. To separate the mandible from whole data, the linked area on each image, such as the TMJ, should be erased, and the patient’s mouth should be kept open by a cotton roll to prevent the dentitions from touching during scanning. The reconstructed mandibular and fibular models are shown in Figure 2.

### Design of defect resection plan, template, and repaired model

**Figure 3:**
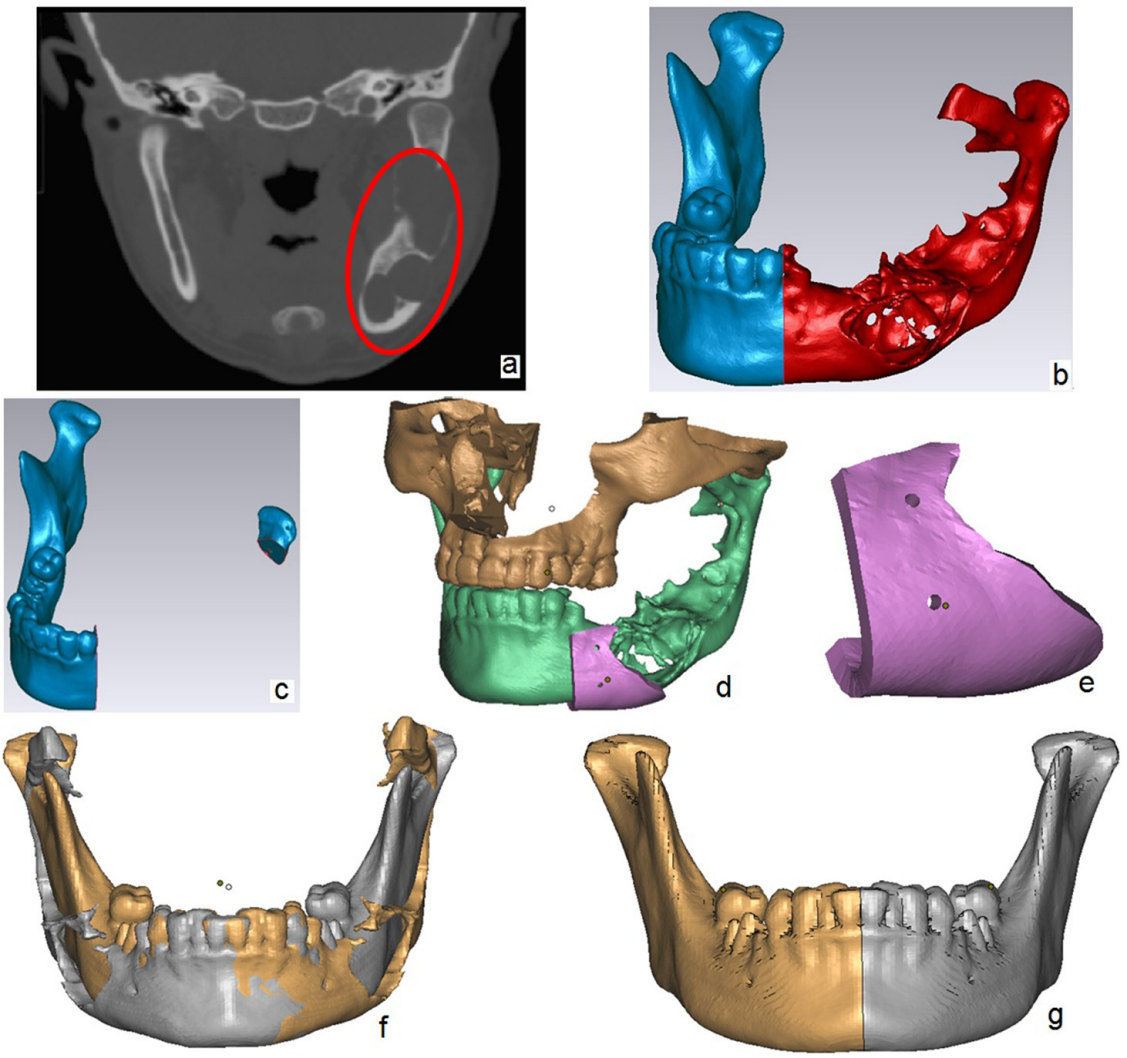
**Design of resection template and repaired model. (a)** CT image with marked defect. **(b)** Resection curves of defect. **(c)** Remain mandible after defect resection. **(d)** Resecting template based on bone surface. **(e)** Resection template. **(f)** Mirrored model and original model of mandible. **(g)** Repaired model after two parts merging.

The original mandibular model is used to design the surgical plan for defect resection and so that the resection curves can be acquired, after which the resection template is designed based on the clearly separated tumor area and resection curves. To separate the tumor defect, the Rhino platform for triangular mesh handling (Rhinoceros, version 5.0, McNeel, Seattle, WA, USA) is adopted to design the resection curves based on the exported STL file of the mandible. To detect the tumor defect boundaries clearly, 2D-resliced images in Mimics can be used as reference, such as the defect area marked by an ellipse shown in Figure 3(a). In this case, the tumor area is from the second premolar in the lower left jaw to the left condyle, and the distance from the border of the ameloblastoma on the bone surface to the internal border is 13.26 mm, so the resection curve is designed between the first premolar and the second premolar, for full excision of the ameloblastoma with size 7.5 cm × 3.5 cm, and volume 19070 mm^3^, as shown in Figure 3(b). After the tumor is excised, the remaining mandible, shown in Figure 3(c), should be linked and repaired with a graft. In mandibular reconstruction, the condyle should ideally be conserved, because it is difficult to restore the TMJ function by a reconstructed condyle from an artificial metal graft or autograft. In this case, the left condyle remained after careful diagnosis and evaluation, although the conserved area was not large, but features whole cartilage over the entire joint head, as shown in Figure 3(c).The template for defect resection is designed via mesh-surface-thickening based on original mandibular model and resection curves, as shown in Figure 3(d). Two holes for template fixation during the operation are designed, and are used to fix the template to the mandible by titanium screws with 2.0-mm diameter. The 3D model for fabrication of the template is shown in Figure 3(e).The repaired model is based on the original model, with the healthy area taken into consideration rather than only the defect area. As shown in Figure 3(f), with Mimics, the mandible is mirrored via an inherent sagittal plane implied in CT data, and is manipulated into ideal position with good fitting of dentition and bone. The mirrored model and original model are then processed in Rhino by plane cut. The defect side is substituted by the healthy side from the central incisor, rather than from the second premolar (i.e.., the defect border), to achieve a more ideal maxillofacial structure and better quality of the repaired model, as well as more convenient manipulation, as shown in Figure 3(g).

### Design of templates for graft resection and shaping

**Figure 4:**
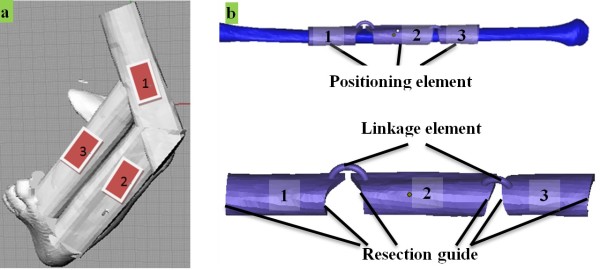
**Design of template for fibular graft osteotomy and harvesting. (a)** Virtual plan for mandibular reconstruction with three barrels of fibular graft. **(b)** Resection template for fibular osteotomy.

The graft resection and shaping plans are also designed on Rhino. For free fibular flap reconstruction, the rules of graft site selection, resection, and shaping are determined by the requirements of harvesting on hard and soft tissue (mucosa or skin) [4]: (1) The vascular pedicle for supplying blood to the graft flap should be carefully maintained during surgery; (2) mandibular bone height should be restored by graft, so a “double-barrel” approach may be adopted; (3) for reconstruction of the mandibular ramus, the third “barrel” is needed, so the resection template should satisfy these requirements; and (4) to restore the patient’s appearance, the graft should be shaped by a shaping template.To harvest an ideal graft, four steps are required: (1) A preliminary plan for graft placement is designed based on the measurement of defect size, and so the length and angle of fibular barrels are initially planned; (2) the fibular model is moved to the mandibular defect area; (3) the fibular model is cut into needed barrels, which are transferred to new positions according to the initial plan; and (4) the positions of the barrels are carefully adjusted, with step 3 repeated if necessary, to obtain an ideal plan for graft shape and placement. In this case, the fibula was cut into 3 segments, 2 of which were placed as double barrels for alveolar bone restoration in the left jaw, with the third segment positioned for mandibular left ramus reconstruction. The 3D model of the final virtual graft is shown in Figure 4(a), which is the basis for template design.For the ideal plan to be translated from computer to clinic, two templates are needed, one for graft resection, and the other for graft shaping and placement. Based on the barrels in the graft plan, the resection template is designed by mesh-surface-thickening on the fibular model and is cut into detached parts along the borders of the barrels. As shown in Figure 4(b), the template parts and enclosed fibular segments, marked 1, 2, and 3, correspond with fibular barrels in the graft plan [Figure 4(a)]. The 3 parts on the template are linked by 2 linkage elements designed to bend shape with flexibility. The placement of the template is realized through fit of the main body (marked as positioning element) on the bone. The guides for bone resection curves are located at both sides of each part, as marked on Figure 4(b).

**Figure 5:**
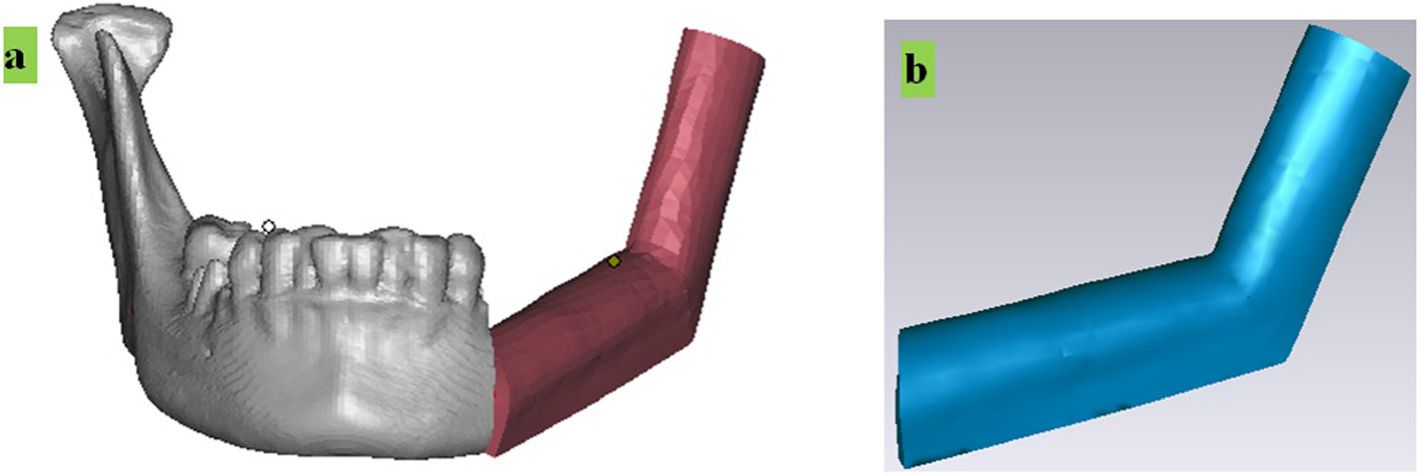
**Design of template for fibular graft shaping. (a)** Shaping template is positioned on mandible. **(b)** Shaping template.

The shaping template is used to place the fibular barrels at correct positions as planned. Based on the virtual graft in Figure 4(a), the shaping template is created through mesh-surface-thickening. Using this template as shown in Figure 5, 3 graft barrels are placed and trimmed to the planned shape, and then fixed by titanium plate with the remaining mandible and condyle *in vivo*.

### Fabrication by 3D printing

Additive manufacturing, or 3D printing as it is popularly known, is a primary method for medical fabrication, and includes the principles of SLA (stereolithography), SLS (selective laser sintering), FDM (fused deposition modeling), etc. A SLS machine, Sinterstation HiQ + HiS^TM^ (3D Systems, Valencia, CA, USA), with broad compatibility for various types of material, and good mechanical properties of the parts produced, was selected to fabricate the oral bone model and surgical templates. The material, DuraForm, is a biocompatible nylon material and satisfies the requirements of USP IV. SLS processing of this material powder was performed by preheating the powder to 70°C and laser-scanning (500-μm focused beam diameter) at 15-W power and 1.257 m/s scan speed. The models and templates were built layer-by-layer using a powder layer thickness of 100 μm, and this process took 5 h. After SLS processing was completed, the models were allowed to cool inside the machine processing chamber for approximately 1.5 h and were then removed and cleaned by compressed air.

### Clinical results

In cooperation with hospitals in Hangzhou, Zhejiang province of China, the proposed method has been in clinical use for 2 years, and more than 15 cases of mandibular reconstruction with template-guided surgery have been performed successfully.

**Figure 6:**
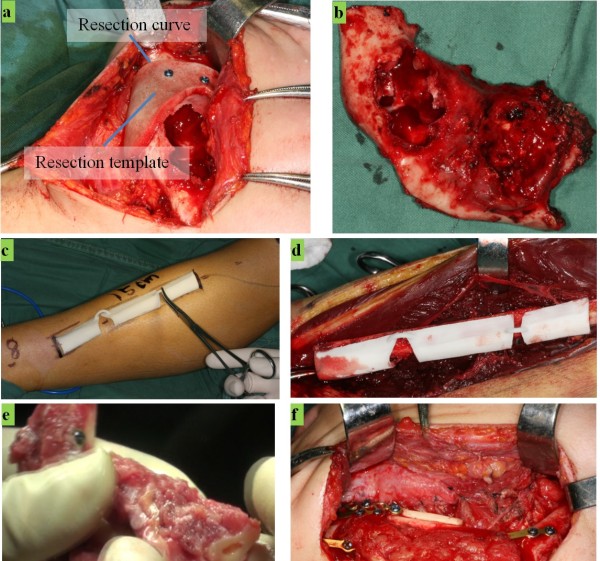
**Fibular graft resection and harvesting. (a)** The template for defect resection is fixed on mandible. **(b)** The resected tumor. **(c)** Surgical routine is drawn on the skin with the help of fibular osteotomy template. **(d)** The fibular osteotomy template is placed on the fibular bone. **(e)** Shaped fibular graft. **(f)** The graft is placed and fixed in the defect area.

**Figure 7:**
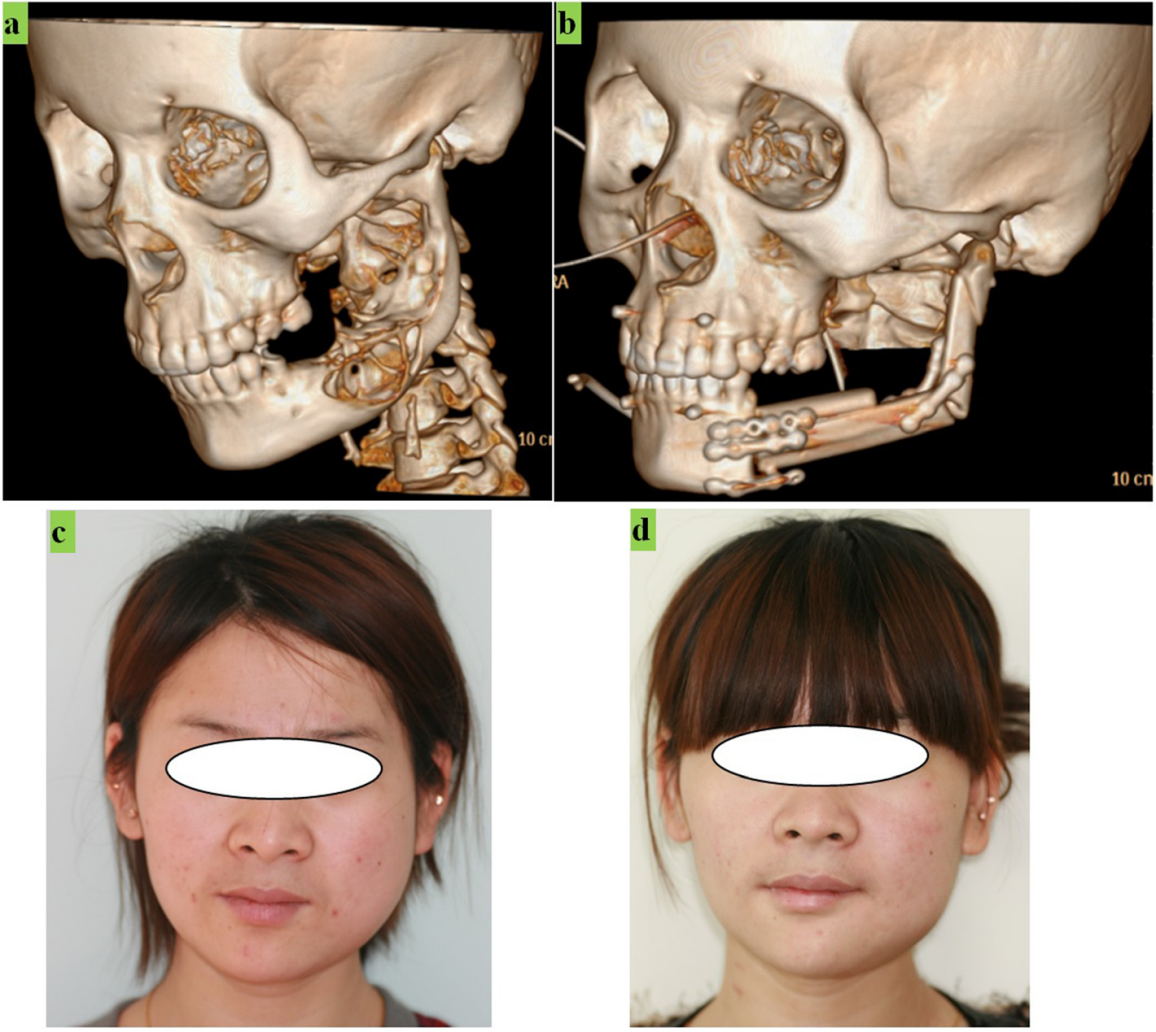
**Comparison of preoperative view and postoperative view. (a)** Preoperative 3D view of maxillofacial bone. **(b)** Postoperative 3D view of maxillofacial bone. **(c)** Photo before surgery. **(d)** Photo of one month after surgery.

For the selected typical case, some typical operations during the clinical course of this case are shown in Figure 6. First, the mandibular defect was opened and exposed, then the resection template was fixed onto bone, and the defect was resected along the resection curve, as shown in Figure 6(a). The separated bone with tumor is shown in Figure 6(b), with measured size 7.5 cm × 3.5 cm and measured volume 19325 mm^3^, which were almost equal to the parameters in virtual plan. Because the left condyle was to be used on the graft, it was kept separate. To prepare the graft, on the left shank, the surgical area was designed and the operative route drawn based on the graft resection template and virtual plan, as shown in Figure 6(c). Then, the fibular flap was exposed and resected as guided by the resection template, then harvested, as shown in Figure 6(d). During fibular graft flap separation, the vascular pedicle of the flap should be handled and cut carefully, since it will be connected vascularly to the mandible by microsurgical vascular anastomosis.According to the predesigned virtual plan, the fibular flap was separated into 3 segments, and then shaped and fixed to a graft. Because the condyle separated from the bone defect would be reused on the graft, it was mounted on the flap at one side. The prepared graft is shown in Figure 6(e). The graft of the fibular flap was placed in the defect area and fixed with the correct mandible by a preformed titanium plate, after which the blood vessels in the graft flap and mandible were connected through microsurgical vascular anastomosis, as shown in Figure 6(f).The results of the surgery are shown in Figure 7. The 3D models of the patient’s mandible before treatment and one week after treatment, reconstructed from CT software, are shown in Figure 7(a) and 7(b). The tumor defect has been replaced by a postsurgical autograft, and the graft has survived, with good blood supply and without obvious bone resorption, for one week. After one month, the patient’s facial aesthetics was restored, as shown in Figure 7(d) compared with the pre-treatment image in Figure 7(c).

The patient’s oral functions, including masticatory performance, mouth opening, and speech, were recovered well. Immediate postoperative mouth opening was 2.6 cm. Jaw movements were painless after one week, and acceptable maxilla-mandibular relationships were obtained without obvious complications. The patient was able to walk at the end of the 1^st^ postoperative month and eat normally. The postoperative scars have healed well and there was no evidence of any infection.

In this case, the entire surgical procedure took approximately 8 h, 20% less than a conventional “free-hand” operation. Obviously, the time used for pre-surgical preparation is increased, including CT scanning, data processing, model reconstruction, and work on design and manufacturing. During surgery, the surgeon does not need to think about the surgical plan; he/she simply executes the predesigned plan under the guide of the templates.

## Discussion

In the illustrated case, the restoration of both function and aesthetics was satisfactory as a result of accurate and efficient surgical procedures. In mandibular reconstruction, most of the existing applications in digital design and manufacturing techniques are still very preliminary. In these applications, the surgical plan is only kept in the computer, rather than realized in the clinic, because no templates are designed and manufactured to guide clinical operations, and only original mandibular and repaired models are fabricated as references [2,7,9,14], or without a defect resection template [26]. In this case, to execute the predesigned plan in the clinic, the templates for defect resection, fibular flap resection, and graft shaping were designed and fabricated, and used in surgery. With the help of templates, the defect can be removed with high accuracy, and an ideal graft can be implanted to restore function and appearance to the patient with a high degree of efficiency and quality.

The fibular flap graft has obvious merits for mandibular reconstruction, including ease of shaping, good blood supply, and sufficient supply of soft and hard tissue. In the past 2 years, 15 cases compatible with fibular grafting were selected for use with the proposed solution, and all achieved satisfactory results.

The most established application of these advanced techniques in dentistry is guided dental implantation. Based on a predesigned plan and surgical guide, accurate surgery can be achieved in the clinic with precise implant placement [10,11]. Compared with mandibular reconstruction, dental implantation is rather easy, and guided surgery can be realized with more facility. Established commercial applications for accurate dental implantation have emerged in recent years, and, worldwide, more than ten companies can supply surgical guide services, notably, NobelGuide^TM^ (Nobel Biocare, Zürich, Switzerland) and Simplant^TM^ (Dentsply, York, PA, USA). Different from artificial implant placement in dental implantation, mandibular reconstruction with an autograft is more complex in operational treatment, which includes defect resection, graft harvesting, and graft placement, so using the solution proposed here for guided dental implantation appears to be rather limited.

Different from existing commercial services, such as SurgiCase^TM^, this solution adopts general platforms for plan and tools design, including Mimics, Geomagic and Rhino, which are easily available and cheaper for customers than special software. As illustrated in clinical case, a total of three templates are used to transfer the virtual plan to clinic and control the operational accuracy, which is an improvement over some research.

To compare the proposed solution to conventional method, we collected 8 cases performed during August 2007 to December 2008 to analysis, which were operated by *free hand* but with preoperative design of virtual plans [27]. The average operational time is saved almost two hours each case with templates, and the accuracy is improved significantly, as listed in Table 2.

**Table 2:** Results comparison

**Variables**	**Templates guided surgery**	**Conventional surgery**
Average operational time	6 h 35 minutes	8 h 20 minutes
Postoperative complications	1 in 15 cases	2 in 7 cases
Average mouth open	3.0 cm	2.8 cm
Accuracy		
Average resection defect size	Plan: 5 × 7 cm	Plan: 4 × 8 cm
	Operation: 5 × 7 cm	Operation: 5 × 9 cm
Average fibular flap length	Plan: 8 cm	Plan: 7 cm
	Operation: 8 cm	Operation: 8 cm

On the surgical field, this solution has the same limitations as the conventional method of fibular flap, so no special limitations exist. But several extra limitations still exist. The first lies in radiology, because the quality of CT image determines the accuracy of the reconstructed model, so the scanner resolution and the scanning parameters should be satisfied, which increase the communication cost between dental surgeon, radiologist, and engineers, and the scanning time is increased too. In China, the radiology department is always very busy, and only some large hospitals own a CT machine with high quality, so getting appropriate data becomes difficult, which limit this solution widely used. Another limitation is preoperative time increasing comparing to conventional operation, because the virtual plan design needs surgeons to discuss and the templates design and fabrication need 3–5 days. The surgeons with abundant experience would not like adopting this solution to save time when they face a lot of patients, and also some patients want to be treated immediately. The third limitation lies in the cost increment, although the cost is lower than other commercial services, but average 3.5 thousands Yuan of extra pay-out is also unaffordable by some patients in China, because it is not included in health insurance.

## Conclusions

To save operating time and improve the effect of surgery on mandibular reconstruction, modern digital techniques, including medical image processing, digital design, and 3D printing, are applied. Based on CT images, a 3D model of the mandible is reconstructed, and the tumor defect can be clearly recognized on the model, which is more precise for diagnosis and graft design. Also, with 3D models of original and repaired mandibles, the graft can be designed conveniently, and thus the fibular segments can be determined. The virtual plan for mandibular reconstruction includes defect resection, fibular segment resection, and graft shaping, which can be clearly communicated to the patient, and is also helpful for young doctors’ study and training. With the help of templates, the predesigned plan can be carried out in the clinic, which guarantees the quality and accuracy of surgery.

Besides the fibular flap, mandibular reconstruction can also be conducted with iliac bone grafts or costochondral grafts. For different grafts, the procedure for planning and template design is similar, based on the same techniques, although some steps are different. Future research will include different graft and error analyses.

## Consent

Written informed consent was obtained from the patient for the publication of this report and any accompanying images.

## Competing interests

The authors declare that they have no competing interests.

## Authors’ contributions

YL performed the whole procedure, and designed the plan. LX participated in template design and fabrication. HZ instructed the plan design and conducted the clinical operation. SL instructed the medical technology and writing. All authors read and approved the final manuscript.
